# Complex geometry of volcanic vents and asymmetric particle ejection: experimental insights

**DOI:** 10.1007/s00445-022-01580-6

**Published:** 2022-07-04

**Authors:** Markus Schmid, Ulrich Kueppers, Valeria Cigala, Donald B. Dingwell

**Affiliations:** Ludwig-Maximilians-Universität (LMU) München, 80333 Munich, Germany

**Keywords:** Explosive eruptions, Vent, Asymmetry, Crater, Gas-particle jet, Eruption dynamic

## Abstract

Explosive volcanic eruptions eject a gas-particle mixture into the atmosphere. The characteristics of this mixture in the near-vent region are a direct consequence of the underlying initial conditions at fragmentation and the geometry of the shallow plumbing system. Yet, it is not possible to observe directly the sub-surface parameters that drive such eruptions. Here, we use scaled shock-tube experiments mimicking volcanic explosions in order to elucidate the effects of a number of initial conditions. As volcanic vents can be expected to possess an irregular geometry, we utilise three vent designs, two “complex” vents and a vent with a “real” volcanic geometry. The defining geometry elements of the “complex” vents are a bilateral symmetry with a slanted top plane. The “real” geometry is based on a photogrammetric 3D model of an active volcanic vent with a steep and a diverging vent side. Particle size and density as well as experimental pressure are varied. Our results reveal a strong influence of the vent geometry, on both the direction and the magnitude of particle spreading and the velocity of particles. The overpressure at the vent herby controls the direction of the asymmetry of the gas-particle jet. These findings have implications for the distribution of volcanic ejecta and resulting areas at risk.

## Introduction

Explosive volcanic eruptions eject gas and pyroclasts at high velocity and temperature into the atmosphere. The related threat to life and infrastructure is a consequence of the eruption’s style and magnitude. In proximal areas (tens of metres to few kilometres), volcanic ballistic projectiles can inflict injury and destruction of property (e.g. Alatorre-Ibargüengoitia et al. 2016; Blong 2013; Williams et al. 2017). Pyroclastic density currents (PDCs) pose an additional risk threatening thousands of lives, agricultural land and farm stock, as well as infrastructure (e.g. Blong 2013; Charbonnier et al. 2013; Druitt 1998; Lube et al. 2020; Sulpizio et al. 2014). Therefore, there is an increasing need for detailed and customised hazard and risk assessment in areas where social and economic activities might be compromised (Fitzgerald et al. 2014).

In recent years, significant advances have been made in monitoring and forecasting of volcanic eruptions (e.g. Dempsey et al. 2020; Johnson et al. 2018; Layana et al. 2020). Yet, unforeseen or larger-than-expected eruptions still claim many human lives. While it would be the safest option to draw large exclusion zones around (potentially) active volcanoes, this is often not socially feasible. In the absence of such measures, achieving a better understanding of source conditions that lead to hazardous explosive eruptions and control the travel distance of volcanic bombs is central to the development of probabilistic hazard maps. Here, we perform rapid decompression experiments and empirically correlate the ejection characteristics of gas-particle jets in the near-vent region with complex vent geometry.

In the near-vent region, volcanic explosions are typically manifested by multiphase underexpanded (pressure at the vent (P_vent_) > atmospheric pressure (P_atmosphere_)) starting jets (Carcano et al. 2014; Kieffer and Sturtevant 1984; Woods and Bower 1995). In nature and in laboratory experiments, vent geometry exerts a prime control on the ejection of gas and gas-particle flows by affecting ejection velocity (e.g. Cigala et al. 2017; Kieffer 1989; Ogden 2011; Wilson and Head 1981; Wilson et al. 1980), jet radius (e.g. Jessop et al. 2016; Koyaguchi et al. 2010; Woods and Bower 1995), jet inclination (Schmid et al. 2020) and gas and gas-particle spreading (Cigala et al. 2021). Furthermore, vent geometry influences the trajectories of volcanic ballistics (Dürig et al. 2015) and the likelihood of column collapse (Jessop et al. 2016). Whether an eruption column collapses or rises as a buoyant plume is governed by the efficiency of entrainment of ambient air (Woods 2010). Factors promoting a buoyant plume are narrow vents, high exit velocity, high gas content and possibly high pressure ratio at the vent (Valentine 1998). The effect of vent shape on flow dynamics has been investigated for vents with radial or axial symmetry [e.g. Deo et al. 2007; Glaze et al. 2011; Jessop et al. 2016; Mi et al. 2004]. To date, the natural complexity of volcanic vents is often greatly simplified in experiments and models, where the vent is commonly treated as a symmetrical circular feature. In reality, volcanic vents are likely complex, with highly asymmetric shapes that can change on short timescales. During the third pulse of the Upper Te Maari eruption in 2012, Breard et al. (2014) reported a sub-vertical jet angled towards the north and a ballistic clast distribution in the northern sector. At the time of the eruption, the Upper Te Maari crater was described as being clearly opened to the north (Breard et al. 2014; Lube et al. 2014). Additionally, preferential emplacement directions of PDCs have indeed been explained by the asymmetry of vents and/or craters (Cole et al. 2015; Lagmay et al. 1999; Major et al. 2013). In these cases, the craters were described as notched or opened to one side causing inclined jets. As a result, PDCs were directed towards the notched or open sides. Here, we explore the relationship between vent asymmetry and asymmetric gas-particle jets.

The near-vent characteristics of volcanic jets are important for our quantitative understanding of volcanic eruptions since they are the first observable manifestation of the related sub-surface processes. Jet attributes directly above the vent derive from the combination of initial conditions and vent geometry, while subsequently, atmospheric conditions (wind field, temperature, humidity) can substantially alter the jet dynamics.

Multiphase jets result from magma fragmentation following deformation and gas expansion and occur over a wide range of eruption styles, e.g. Strombolian, Vulcanian and Plinian eruptions (Gouhier and Donnadieu 2011; Koyaguchi and Woods 1996; Scharff et al. 2015; Taddeucci et al. 2012). Thus, in general, the complex interactions between the ejected phases and their characteristics (e.g. gas-particle ratio) exert strong controls on the dynamics of the jets. Interdependencies between the fluid phase (gas, melt and vapour) and solid particles have been reported for two-way (e.g. Bercovici and Michaut 2010; Burgisser et al. 2005; Carcano et al. 2014; Cerminara et al. 2016) and four-way coupling. The degree of coupling between the solid and the gas phases significantly affects particle acceleration and resulting trajectories.

Magma fragmentation and volcanic jet generation have been successfully mimicked in shock-tube experiments (e.g. Alidibirov and Dingwell 1996; Arciniega‐Ceballos et al. 2015; Cigala et al. 2017; Kueppers et al. 2006b; Montanaro et al. 2016) and such scaled laboratory experiments are a key to exposing initial conditions of volcanic eruptions that are beyond direct observation. Cigala et al. (2017) empirically correlated the temporal evolution of particle exit velocity from radially symmetric vents with internal vent geometry, particle load, grain size distribution, conduit length and temperature. High-speed video footage of these experiments was used to analyse the temporal evolution of the angular deviation of particles from the vertical (Cigala et al. 2021).

Whereas in nature, gas-particle ejection and jet inclination might be influenced by inclined conduits (Zanon et al. 2009), debris coverage (Capponi et al. 2016), variable explosion depth (Dürig et al. 2015; Salvatore et al. 2018), pre-existing craters (Graettinger et al. 2015; Taddeucci et al. 2013) or an inhomogeneous high-viscosity layer (Kelfoun et al. 2020). In this study, we can exclude all of these factors and investigate the sole effect of subsurface initial conditions (pressure, particle size and density) and vent geometry.

**Fig. 1:**
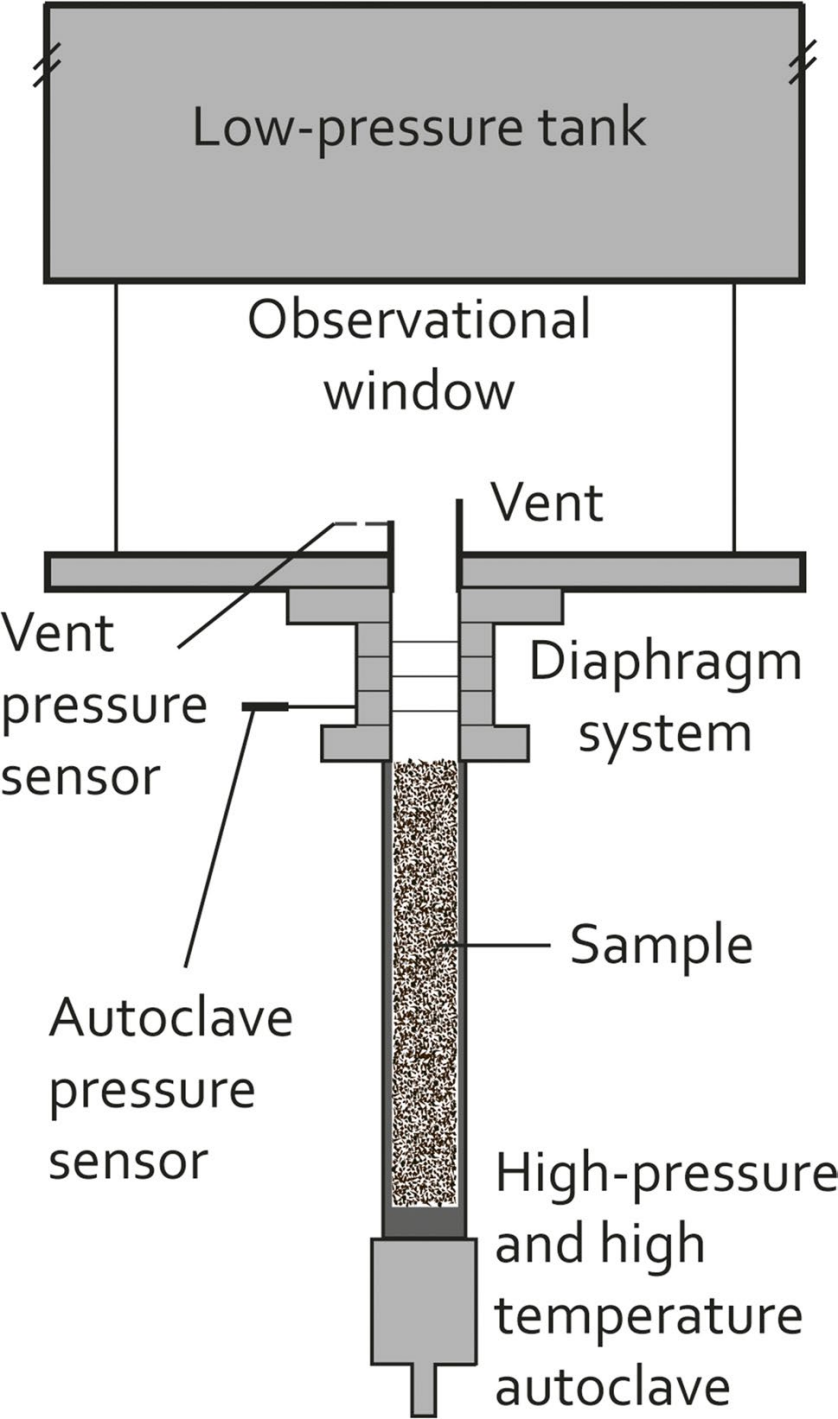
Shock-tube setup at Ludwig-Maximilians Universität, Munich with a high-pressure/temperature autoclave including samples, the diaphragm system and the low- pressure section above the vent

**Fig. 2:**
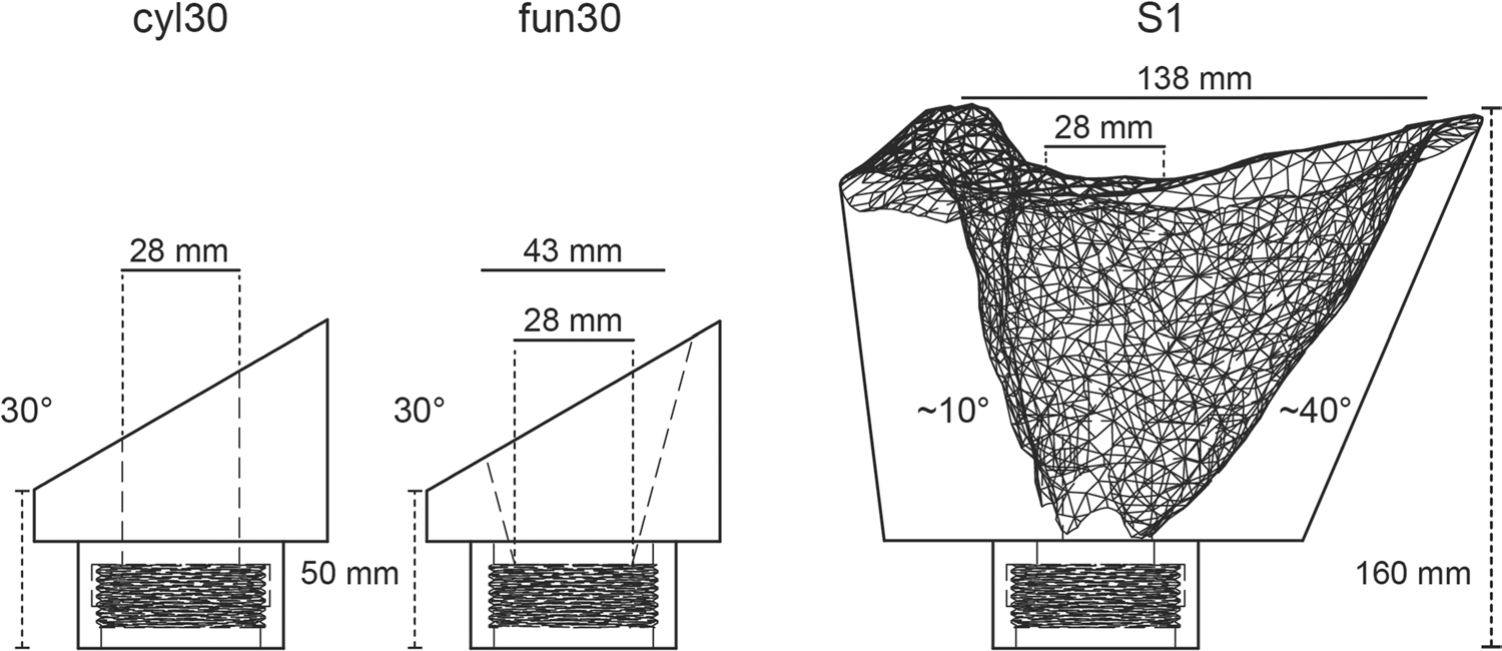
Sketch of the three vent geometries used for the present study. They can be distinguished by their characteristic geometry element with a slanted exit plane (*cyl30* and fun 30) and a variable divergence angle (*S1*)

Building on the two vent geometries of Cigala et al. (2017) (cylindrical and 15° diverging inner geometry (funnel)), we increased the complexity of vent geometry by introducing variably slanted surface planes (5°, 15°, 30°) to investigate the gas ejection from six axisymmetric vent geometries (*cyl05*, *cyl15*, *cyl30*, *fun05*, *fun15*, *fun30*; Appendix Fig. 8) at four starting pressure ratios that, in previous experiments without particles, revealed asymmetric spreading angles and inclined gas jets (Schmid et al. 2020). Here, we performed repeatable shock-tube experiments (Fig. 1) with three vent geometries (*cyl30*, *fun30*, *S1*, see Fig. 2 and Appendix Fig. 8), two types of particles (scoriaceous and pumice), each with three particle size classes (0.125–0.25, 0.5–1, 1–2 mm) and two experimental pressures (8 and 15 MPa) to further elucidate the effect of vent geometry and gas-particle coupling on the ejection of a gas-particle mixture.

## Materials and methods

### Experimental setup

The shock-tube setup used in this study is an evolved version of the “fragmentation bomb” developed by Alidibirov and Dingwell (1996) that has been adapted and utilised in many studies to date (e.g. Arciniega‐Ceballos et al. 2015; Cigala et al. 2017; Kueppers et al. 2006a, 2006b; Montanaro et al. 2016; Spieler et al. 2004). Here, we used the latest version, including the modifications introduced by Cigala et al. (2017) (Fig. 1).

The setup consists of a high- and low-pressure section, separated by diaphragms. Two copper diaphragms (each will break at ~ 4.6 MPa) or three iron diaphragms (each will break at ~ 6.1 MPa) were used for the incremental pressurisation to the final autoclave pressure of 8 and 15 MPa, respectively. The autoclave (*Nimonic 105* alloy) has an internal diameter of 28 mm and a volume of 127.4 cm^3^. Upon intended failure of the uppermost diaphragm, the diaphragm(s) below go outside their stability field, open, and pressure equilibration initiates. The associated rapid decompression of the autoclave allows the gas to expand. The associated gas flow accelerates the particles; the gas-particle mixture is ejected through a vent into the low-pressure section, a 3 m high stainless-steel tank at ambient conditions, sitting above a 35 cm high transparent Perspex cylinder.

Three different vent geometries were used in this study, with increasing “topographic complexity” based on the findings of Cigala et al. (2017) and Schmid et al. (2020). At the base, all are the geometrical extension of the underlying autoclave (inner diameter of 28 mm). Two vent geometries were fabricated from *1.4305 NiCr* steel. They have already been used by Schmid et al. (2020), where they showed the biggest impact on gas-jet dynamics. They have bilateral symmetry with a slanted top plane (30° inclination) above a cylindrical (*cyl30*) or 15° diverging funnel (*fun30*) inner geometry, respectively. Accordingly, lateral gas expansion can already start below the vent exit for the diverging vent. The lower vent exit height was always 50 mm (Fig. 2). The top exit of those vents was 16 and 30 mm higher, respectively. The third vent (*S1*) resembles the geometry of the active S1 vent on Stromboli in May 2019. All vents are not eroded by the jet during experiments and are re-used multiple times.

During the May 2019 field campaign, aerial imagery was collected by unoccupied aerial vehicle (UAV) of a specific vent (called S1) and subsequently, a 3D model was created by Structure from Motion (SfM) photogrammetry using *Agisoft Metashape*. For a detailed description of the field campaign and the processing, refer to Schmid et al. (2021). The created 3D mesh was transformed into a printable body with *Autode*sk *Fusion 360*. Afterwards, the outer shape of the vent was designed and exported as Standard Triangle Language (STL) file, a standard file format used in 3D printing. The software *Slic3r* was used to convert the STL file into printing instructions (G-code) for the 3D printer by cutting the model into horizontal slices (layers) and the required toolpaths to form the 3D printed model. A *Renkforce RF1000* 3D printer that was controlled by the *Repetier-Host* software was used. The model was printed with *polylactic acid* (PLA) filament with a 0.5 mm nozzle, a layer thickness of 0.4 mm and 60% infill density in a honeycomb structure. The printed vent was fixed to a steel vent mount to withstand the applied experimental conditions resulting in a total height of ~ 160 mm. The inner diameter up to the throat of the vent is 28 mm as in the other vents (Fig. 2). The average diameter at the vent exit is 138 mm compared to 28 mm (*cyl30*) and 43 mm (*fun30*). The defining geometry element of the S1 vent was the asymmetric divergence with ~ 10° on one side and ~ 40° on the opposing side.

Two pressures (8 and 15 MPa) and six different samples were tested for each vent geometry. The pressures have been selected to allow a direct comparability with earlier studies on gas-particle and gas-only jets with variable vent geometry that have been performed at 5, 8, 15 and 25 MPa (Schmid et al. 2020). We used two types of natural samples from the East Eifel volcanic region (Germany): scoriaceous fragments of a porous lava flow (SL) and pumice particles from the Laacher See eruption (LSB). Three particle sizes were used for both types: (1) fine, 0.125–0.25 mm; (2) medium, 0.5–1 mm; and (3) coarse, 1–2 mm. The average density was 2.5 g/cm^3^ and 1.4 g/cm^3^ for the scoria (SL) and the pumice (LSB), respectively (Douillet et al. 2014). The particle load for all experiments was between 38 g (LSB 1–2 mm) and 175 g (SL 0.125–0.25 mm) (Table 1). All experiments were conducted at ambient temperature (~ 25 °C) with argon as pressurising gas.

**Table 1:** Average sample load for all particle types and particle size fractions. The particle-vent ratio is calculated by dividing the medium particle size of each particle size fraction by the basal vent diameter (28 mm)

Particle [mm]	Sample load [g]	Particle/conduit ratio
SL		
0.125–0.25	175	0.007
0.5–1	152	0.027
1–2	143	0.054
LSB		
0.125–0.25	54	0.007
0.5–1	44	0.027
1–2	38	0.054

Once the experiments were initiated, the instantaneous pressure drop in the autoclave (> 1 GPa/s, Spieler et al. (2004)) was recorded by a static pressure sensor (*KISTLER 4075A500*) at the top of the autoclave to trigger the recording system. We recorded the experiments with a high-speed camera (*Phantom V711*) and a pressure sensor (*KISTLER* 601A) at the vent exit. All experiments were filmed at 10,000 frames per second (fps) and a resolution of 1280 × 600 pixels (*cyl30* and fun 30), 864 × 760 pixels (*S1*, 15 MPa) or 960 × 704 pixels (*S1*, 8 MPa). The field of view was approximately 27 × 13 cm, 24 × 18 and 25 × 19 cm, respectively (~ 240 µm/pixel). The camera was aligned orthogonally to the symmetry plane of the vent and centred on the vent axis. The experiments were analysed over a duration of 15–20 ms. Subsequently, particles continued to leave the vent but the overpressure in the autoclave had been exhausted.

**Fig. 3:**
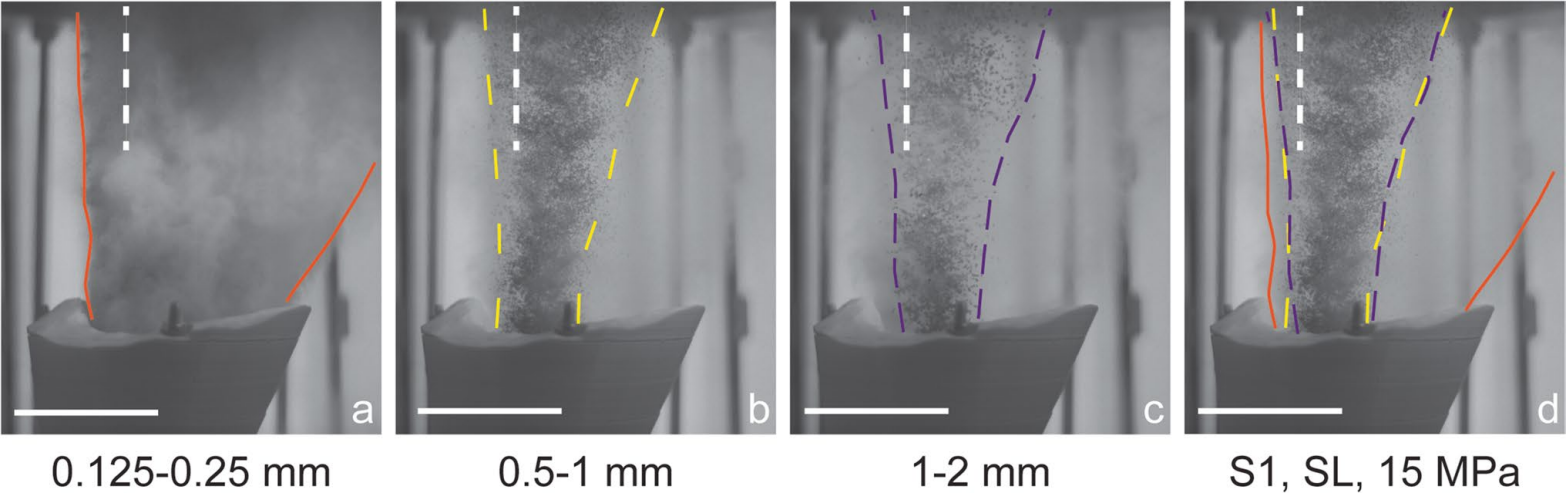
For a qualitative comparison of the various experimental conditions, the outlines of the jets were manually traced in single frames at specific times after the first gas became visible (t_0_). The underlying image for the stacked version in panel **d** is always the single frame of 0.5–1 mm experiment. Here shown for experiments performed with vent geometry *S1*, SL particles, room temperature and 15 MPa starting pressure. The scale bar shows 10 cm. The same colour coding of the contours was used in Fig. 5 and Appendix Fig. 10. The dotted white lines mark the centreline of the vents

We exported scaled single frames to manually and optically analyse them with the *Fiji* (Schindelin et al. 2012) plugin *MTrackJ (*Meijering et al. 2012*)*. We measured the particle spreading angle of the gas-particle jets and the particle ejection velocity. The spreading angles reported here are always the maximum spreading angle that was reached during a single experiment. The particle spreading angle is the angular outward deviation from the vertical continuation of the inner autoclave walls. It was measured as a tangent along the edge of the gas-particle jet starting at the vent exit to the upper limit of the field of view. All angles reported below represent averaged values from three repeated measurements. For a qualitative comparison of the temporal evolution of the gas-particle jets and the asymmetry of particle spreading angle, the jet boundary (as defined by the presence of particles) was traced for each experimental condition and subsequently stacked (Figs. 3, 5 and Appendix Fig. 10). The particle velocity is measured between 1 and 2 ms after the first particle ejection (t_0_). Each particle was tracked over five still frames, and the average velocity is given for > 25 particles.

The reproducibility of experiments in this experimental setup has been demonstrated in several studies (e.g. Alatorre-Ibargüengoitia et al. 2011; Cigala et al. 2017; Kueppers et al. 2006b). The heterogeneity of natural samples has a big influence on the fragmentation behaviour of the sample. In the present study, loose particles were used that were accelerated with negligible preceding fragmentation. We repeated selected experiments to test reproducibility and experiments influenced by irregularities in the experimental procedure (e.g. imperfect opening of the diaphragms). The reproducibility of gas and gas-particle jet spreading angles was demonstrated by Cigala et al. (2021) and Schmid et al. (2020). The subjective error by optically and manually measuring the spreading angles was quantified by letting three individuals analyse the same experiment and comparing the results (Cigala et al. 2021). Since the measuring methodology in the present study is the same and the same experimental setup was used, we assume negligible operator subjectivity as well. We tested the reproducibility of particle ejection velocity by repeating individual experimental conditions three times and analysed each experimental run three times each (min. 25 representative particles in each experiment). We found that the variance in particle ejection velocity within each experiment is higher than the variance between the repetition experiments (Fig. 9). The standard deviations of three measurements (25 particles each) of the same experiment at a given time were up to 17 m/s. In contrast, comparing the average velocity of the three experiments with identical starting conditions had a 5 m/s standard deviation.

### Scaling

For the experiments presented here, the same non-dimensional scaling was applied as for the experiments of Cigala et al. (2017) and Schmid et al. (2020). This manner of scaling has been proven suitable for rapid decompression experiments (e.g. Dellino et al. 2014; Dioguardi et al. 2013) because two explosions at vastly different scales, e.g. in nature and the laboratory, are equivalent if all non-dimensional parameters match.

We calculated Reynolds number (Re), Mach number (M) and the Stokes number (St) to describe the fluid flow dynamics and the coupling between gas and particles. The reference quantities of Re and M were calculated following the one-dimensional isentropic theory (Oswatitsch 1952) by estimating gas density, viscosity and flow velocity based on the starting experimental conditions. We stress that the experiments performed here are highly dynamic, and the values listed in the following are maximum values (Table 2). Schmid et al. (2020) calculated Re for these experiments at characteristic flow conditions, e.g. at the throat of the vent, at the vent exit and fully expanded flow conditions above the vent. For the *cyl30* vent, Re was between 3.58 × 10^7^ (8 MPa, throat) and 2.21 × 10^8^ (15 MPa, fully expanded), and for the *fun30* vent, it was between 3.58 × 10^7^ (8 MPa, throat) and 3.40 × 10^8^ (15 MPa, fully expanded). Re for the *S1* vent was calculated at 1.90 × 10^7^ (8 MPa, throat) and 1.09 × 10^8^ (15 MPa, fully expanded). In volcanic eruptions, Re can be between 10^5^ and 10^8^ (Clarke 2013) or as high as 10^11^ (Kieffer and Sturtevant 1984).

**Table 2:** Maximum non-dimensional numbers calculated for the *cyl30*, *fun30* and *S1* geometry at 8 and 15 MPa experimental pressure. Mach number (*M*) was calculated at the lower vent exit height (*cyl30* and *fun30*) and the *S1*’s average exit diameter. Reynolds number (Re) was calculated at the throat of the vent, the vent exit height (lower side for *cyl30* and *fun30*) and fully expanded conditions. The characteristic length used to calculate these values is the vent exit diameter (28 mm for the cylindrical vent, 43 mm for diverging vents and 138 mm for *S1*). St was calculated for both sample types, each with a particle size of 0.5–1 mm and 1–2 mm

Pressure	*M*	Re			St			
[MPa]	Exit	Throat	Exit	Fully expanded	SL		LSB	
cyl30					0.5–1 mm	1–2 mm	0.5–1 mm	1–2 mm
8	1.6	3.6E + 07	4.8E + 07	6.8E + 07	26	45	20	35
15	1.6	6.7E + 07	9.7E + 07	2.2E + 08	17	31	17	26
fun30								
8	3.2	3.6E + 07	9.5E + 07	1.0E + 08	17	33	13	26
15	3.5	6.7E + 07	2.5E + 08	3.4E + 08	12	22	8	17
S1								
8	10.5	1.9E + 08	6.2E + 08	3.3E + 08	5	10	3	14
15	14.3	3.6E + 08	3.6E + 09	1.1E + 09	3	6	2	9

M is the dimensionless quantity for the ratio between fluid velocity and the speed of sound of the surrounding media. It was calculated by following Saad (1985) to be 1.6 for the *cyl30* vent and 3.2 or 3.5 for the *fun30* vent at 8 MPa and 15 MPa, respectively (Schmid et al. 2020). The *S1* vent with an exit diameter of 138 mm has a M of 10.47 (8 MPa) and 14.31 (15 MPa). Volcanic eruptions frequently exhibit jets with M > 1 (Kieffer and Sturtevant 1984).

The Stokes number (St) is the particle’s momentum response in relation to the surrounding flow field, i.e. it describes how well a particle couples to the flow. We calculated St for fully expanded conditions for experiments with 0.5–1 mm and 1–2 mm (of SL and LSB) particles following Carcano et al. (2014). The maximum velocity of gas and particles is required as an input to calculate St. While it was possible to measure the particle velocity for experiments with 0.5–1 mm and 1–2 mm particles, it was not possible to determine the velocity of individual particles in experiments with 0.125–0.25 mm particles. In addition, it was not possible to determine reliable gas-velocity in this experimental setup (Cigala et al. 2017), and we had to revert to theoretical values following one-dimensional isentropic theory (Saad 1985; Woods and Bower 1995). For the range of particle size (0.5–1 mm and 1–2 mm), particle densities, ejection velocities and vent diameters used in the present study, St was between 45 (scoria, 1–2 mm) and 2 (pumice, 0.5–1 mm). Theoretical investigations suggested that particles with St > 1 are not coupled to the carrier gas-phase (Carcano et al. 2013, 2014; Woods and Bower 1995). The 0.125–0.25 mm particles should accordingly be better coupled to the gas-phase in our experiments as their St is close to 1 (Cigala et al. 2017).

By using 3D printing to produce the *S1* vent, we introduced surface roughness into the system. Based on the findings of Alsoufi and Elsayed (2017), we estimated the surface roughness for our vent to be between 0.045 and 0.071 mm. For fluid flows with high Re, the wall friction depends solely on the friction factor, a ratio of wall irregularity size to conduit size (Wilson et al. 1980). Here, the friction factor is 0.0016–0.0026 at the top of the conduit and 0.0005–0.0003 at the vent exit. Wilson et al. (1980) stated that the calculated range of friction factors for natural conduits varies for most cases between 0.005 and 0.02. Hence, we assume that the roughness related to the 3D printing process has minor influence compared with natural conduit roughness.

Given the broad range of particle sizes of pyroclasts emitted by volcanic eruptions and the similarity of Re, we suggest that our experiments reproduce well the dynamics of volcanic eruptions for gas-particle jets in different St regimes.

## Results

### Particle spreading angle in gas-particle jets

**Fig. 4:**
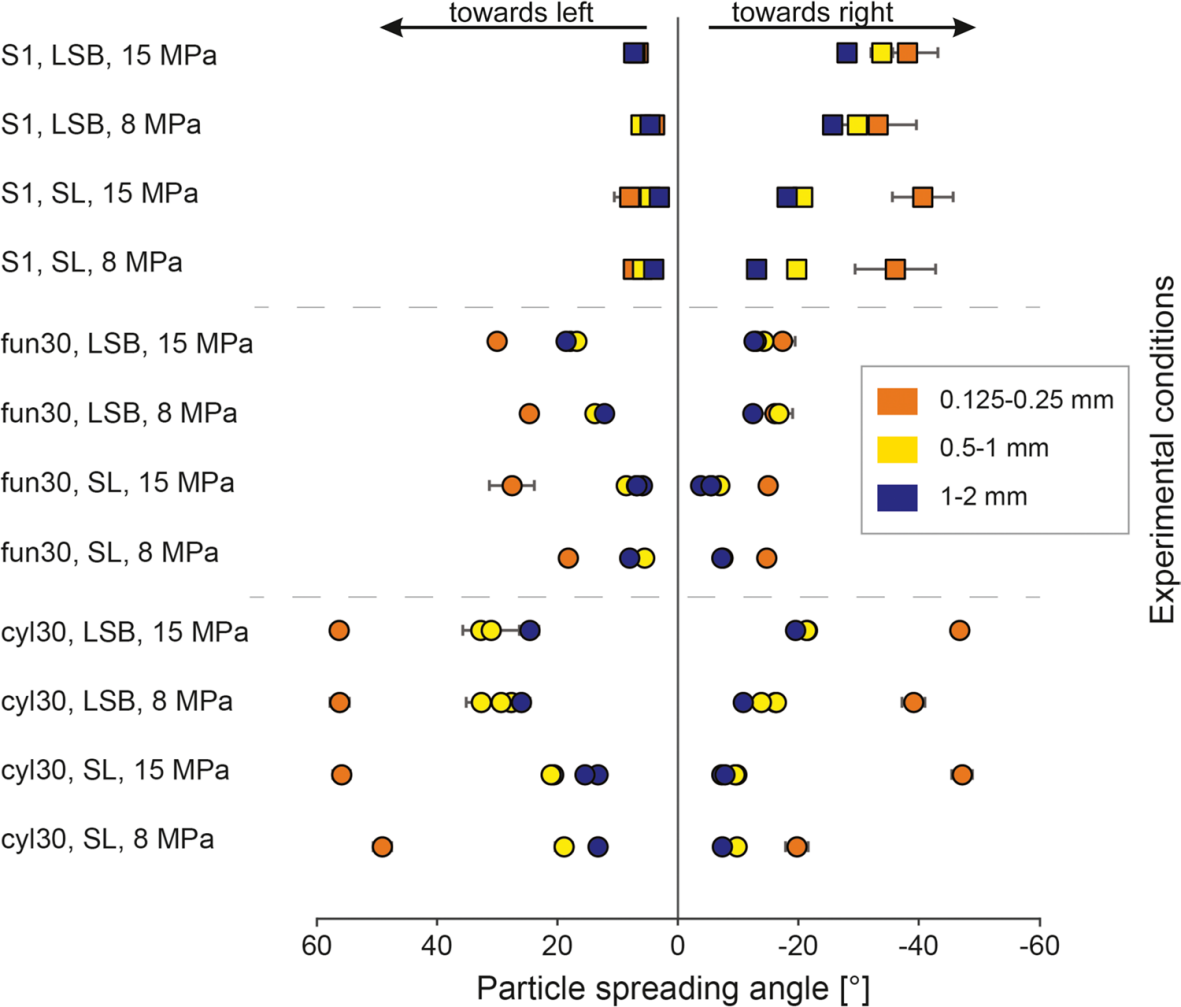
Maximum particle jet spreading angles plotted for all experimental conditions. Positive values are spreading angles on the left side of the vent, negative values on the right side. Circular symbols represent experimental runs with the *cyl30* and fun 30 geometry, square symbols for *S1*. The colours represent the particle sizes of 0.125–0.25 (orange), 0.5–1 (yellow) and 1–2 mm (blue). Error bars represent the standard deviation for the average of three repetitions of measurements. Error bars can be smaller than the associated symbol

In experiments with identical conditions, the *cyl30* vent showed a higher maximum particle spreading angle than the *fun30* vent (Fig. 4). This difference was especially pronounced on the left (lower) vent side. For all experimental runs, particle size had the biggest impact on jet spreading angle, where the fine particles consistently showed the largest particle spreading angle. In all cases, experiments with *cyl30* and *fun30* geometries exhibited an asymmetric jet spreading angle with a larger maximum spreading angle on the left (lower) vent side than on the right side. In experiments with the *fun30* vent, the maximum spreading angle at the beginning of the gas expansion showed a larger spreading angle on the lower vent side. However, throughout the remaining duration of the experiment, the larger spreading angle was observed on the higher vent side. For experiments with the *cyl30* geometry, a larger spreading angle was always measured on the lower vent side.

The jet spreading angle measured for the *S1* vent could not be directly compared with the *cyl30* and *fun30* vent geometries because of the difference in the vent exit height and the resulting offset in time. However, the spreading angle of the jet emitted through the *S1* vent was also asymmetrical, with larger spreading angles on the right (more divergent) vent side than on the left side. The spreading angle measured at the steep side of the vent was small and seemed relatively unaffected by particle size, density or experimental pressure.

In general, experimental pressure was positively correlated, and particle size and density were negatively correlated with gas-particle jet spreading angle. The vent geometry exerted the strongest control and governed the direction and degree of particle spreading angle asymmetry, manifested in visually inclined jets. The effect of particle size was strongest for the fine particle size fraction (Fig. 3a and d). In contrast, the difference between medium and coarse particles was less distinctive and depended on vent geometry. Particle density visibly affected gas-particle jet dynamics with an inverse correlation between larger particle spreading angles and density (larger spreading angles for LSB particles than for SL particles). The magnitude of this difference varied with particle size and pressure. All other experimental conditions constant, 15 MPa pressures were generally correlated with a larger particle spreading angle than 8 MPa pressure. The only exception was *S1*’s left (less divergent) side, where the spreading angle was seemingly unaffected by changing initial conditions.

**Fig. 5:**
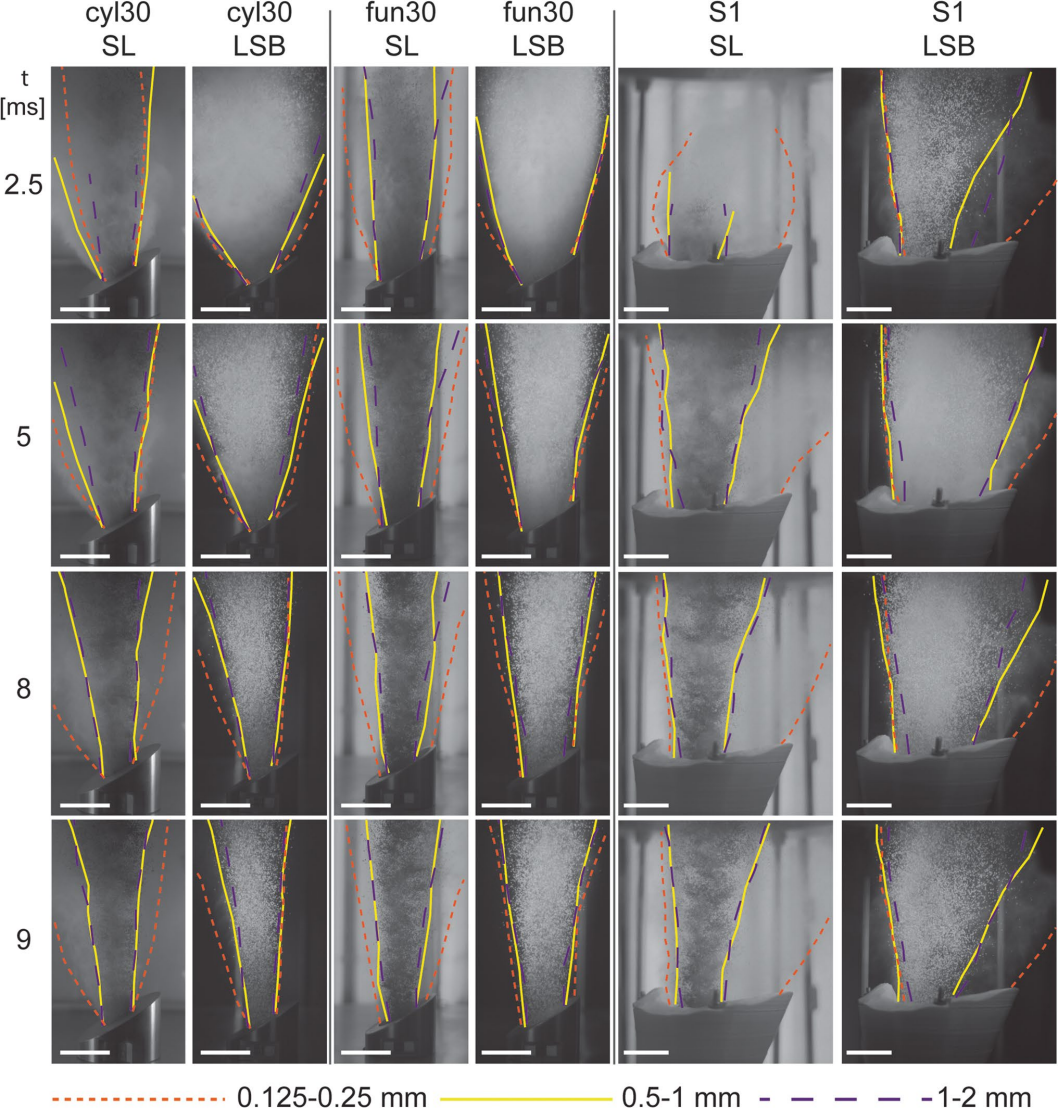
Time series of jet spreading angles at 15 MPa experimental pressure. Each vertical column shows the temporal evolution of particle ejection, with four rows at 2.5, 5, 8 and 9 ms after the onset of the gas ejection. The columns represent six different experimental conditions, using particles of two different densities (SL, LSB) and 3 different vent geometries (*cyl30*, *fun30*, *S1*). Colour lines (see Fig. 3 for explanation) mark the outlines of particle ejection. For every individual stack of images, the corresponding image from the experiment with 15 MPa and 0.5–1 mm particles was taken as basis. *t* = 0 is defined as the onset of gas ejection and the scale bar shows 5 cm. This figure was created as in Fig. 3. Appendix Figure 10 shows the series with 8 MPa experiments

Figure 5 shows the temporal evolution of the gas-particle jets and the asymmetry of particle spreading angles as a function of particle size and vent geometry. All experiments exhibited the largest spreading angle at the beginning of the experiments. With proceeding decompression, spreading angle decreased. Fine and light particles showed a larger spreading angle that could be maintained longer than for coarse and dense particles. In the beginning, 2.5 ms after the first gas ejection, the gas-particle jet emitted by the *fun30* geometry (see Fig. 5, central column) was inclined towards the left (lower) vent side. At *t* = 5 ms, and later in the experiment, the jet was inclined towards the opposite side, the right (higher) vent side. In experiments with the *S1* geometry, the particle spreading angle was sub-vertical on the left side of the vent for all experimental conditions and at all time steps. On the right (more diverging) side of the vent, larger spreading angles were observed than on the left vent side. The *S1* geometry had a higher vent exit height than the other geometries, which caused a delayed ejection of SL particles visible at *t* = 2.5 ms, while LSB particles filled the entire field of view (Fig. 5, top row). The delay was even more apparent in the 8 MPa experiments (see Appendix Fig. 10). Towards the end of the experiments, when the overpressures in the autoclave was exhausted, particles were still ejected but without a directional bias.

### Particle ejection velocity

**Fig. 6:**
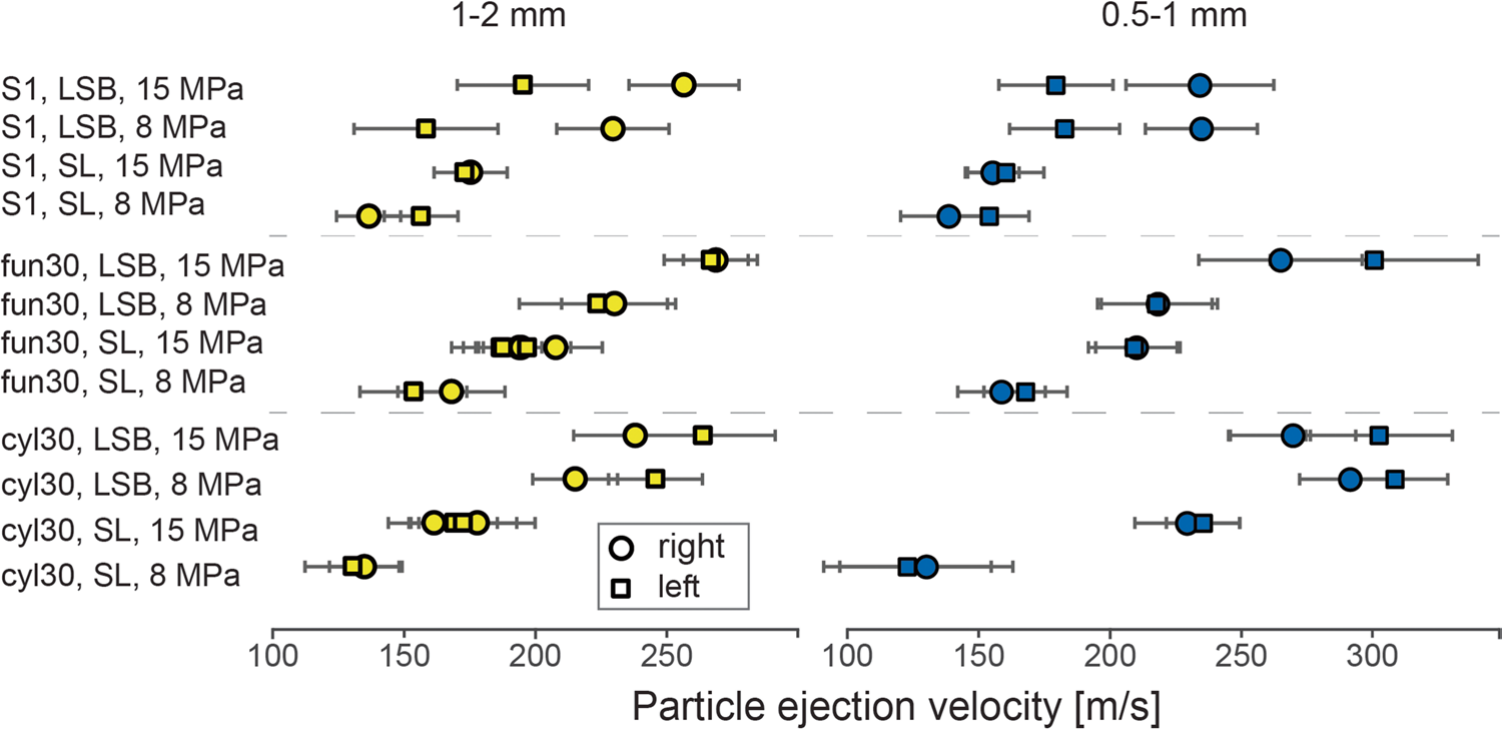
Maximum particle ejection velocity plotted for all experiments with 0.5–1 and 1–2-mm particles. Dots mark velocities on the right-hand side (higher side of *cyl30* and *fun30* vents and more diverging side of *S1*) of the vent while squares mark velocities on the left side (lower side of *cyl30* and *fun30* vent and less diverging side of* S1*). Error bars represent the standard deviation of the averaged particle velocity

Differences in particle ejection velocity are a function of particle density, vent geometry, pressure and subordinately particle size. For the fine particles, it was not possible to obtain particle ejection velocity because of lack of resolution. Lower particle density (LSB) accounted for up to > 100 m/s (> 200%) higher velocities than SL samples (Table 3, Fig. 6). 15 MPa starting pressure caused increased particle ejection velocity (up to ~ 50 m/s greater velocity, ~ 25%) compared to 8 MPa. Accordingly, the highest ejection velocities were observed at the beginning of particle ejection in LSB particles and 15 MPa overpressure experiments. Usually, the *fun30* vent showed higher particle ejection velocities compared to both other vents (up to ~ 30 m/s). Furthermore, vent geometry caused the asymmetric distribution of particle velocity, i.e. faster particles on one side of the vent. We observed up to ~ 60 m/s velocity difference in experiments with the *S1* vent geometry and LSB particles. The higher velocity was measured on the right (more diverging) side of the vent. In experiments with the *cyl30* and the *fun30* geometry, LSB particles showed a higher velocity on the left (lower) side. In the case of the *cyl30* vent at both 8 and 15 MPa pressure, the *fun30* vent was only at 15 MPa. In experiments with SL particles, no distinctive velocity distribution was observed (Fig. 6). There was no clear correlation between particle size and ejection velocity with a tendency for higher velocities for finer particles. In general, particle velocity varied substantially, even within the same experiment and at the same ejection time.

**Table 3:** Particle ejection velocity for all experimental conditions. The velocity was always measured between 1 and 2 ms after the ejection of the first particles. On each side of the vent, 25 particles were measured and averaged (v_left_ and v_right_). Positive values of Δ_v_ indicate higher velocities on the left vent side. All velocities are in m/s

Experiment	0.5–1 mm			1–2 mm		
	v_left_	v_right_	Δ_v_	v_left_	v_right_	Δ_v_
cyl30						
SL, 8 MPa	131	124	7	135	131	4
SL, 15 MPa	230	236	− 6	169	171	− 1
LSB, 8 MPa	292	309	− 17	215	246	− 31
LSB, 15 MPa	270	303	− 33	238	264	− 26
fun30						
SL, 8 MPa	160	169	− 9	168	154	14
SL, 15 MPa	211	210	1	199	190	8
LSB, 8 MPa	219	219	0	230	223	6
LSB, 15 MPa	266	301	− 36	268	267	2
S1						
SL, 8 MPa	139	155	− 16	136	156	− 20
SL, 15 MPa	156	161	− 5	175	173	2
LSB, 8 MPa	236	184	52	229	158	71
LSB, 15 MPa	235	180	55	256	195	61

## Discussion

Gas-particle jets respond to complex vent geometry by exhibiting asymmetric behaviour regarding jet spreading angle and particle velocities. Experimental vent geometry governed the general direction and behaviour of the gas-particle jets, while particle properties and overpressure controlled how well the particles followed the forcing (jet spreading and inclination) exerted by the vent geometry. Once decompression was initiated, the ensuing expansion led to a vertical gas flow within the autoclave. The related drag accelerated particles, thereby transferring a significant portion of the initially stored energy into kinetic energy. The geometric boundary conditions (conduit length or depth of magma surface and the topography) controlled the velocity and residual overpressure of the gas phase at the transition into the atmosphere. If jets were underexpanded at the vent, the lateral gas expansion will act on all particles.

### Particle spreading angle in gas-particle jets

Vent geometry had the most striking effect on particle ejection dynamics as it caused the largest differences in the particle spreading angle and controlled the asymmetry of the particle jet. Experiments with the *cyl30* geometry showed the strongest horizontal expansion and the highest calculated overpressure at the vent exit. Because of the slanted top of the vent, the lateral expansion started first on the left (lower) side of the vent, while the lateral confinement still prevented expansion on the right (higher) side. As a result, the jet exhibited asymmetrical particle spreading angles with larger spreading on the vent’s left (lower) side. In experiments with the *fun30* vent, the initial gas expansion started inside the vent, thereby partially accommodating overpressure. Consequentially, the spreading angle was smaller than for the cylindrical geometry. Because of the slanted top, the spreading angle was also asymmetric, with a larger angle on the left (lower) side. As the pressure at the vent further decreased, the gas-particle jet changed its direction and exhibited a larger particle spreading angle on the right (higher) vent side after ~ 5 ms. This behaviour was unique for the *fun30* geometry and can be linked to the more efficient decompression and lower vent exit pressure (Table 4). An inclination of the jet towards the higher vent side was observed for gas jets (Schmid et al. 2020) and in numerical models (Lagmay et al. 1999), linked to the transition from underexpanded to overexpanded flow conditions. The *S1* vent showed asymmetric particle spreading angles, although there was no difference in vent exit height. For all experiments with *S1*, particle trajectories on the left side seem to be a geometric extension of the inner vent wall. This likely indicates that gas overpressure had been accommodated before reaching the vent exit height. On the right side of the vent, the strong divergence allowed lateral spreading of the particles as a result of a non-uniform gas expansion.

Both particle size and density influenced the degree of coupling between a particle and the surrounding expanding gas flow. Accordingly, the additional lateral expansion of the gas phase above the vent visibly manifested as particle spreading angle. Overall, particle size was negatively correlated with particle spreading angle, and the fine particles always exhibited the largest values. Owing to their lower bulk density, LSB particles were better coupled to the gas than the denser SL particles and generally showed larger spreading angles. This was especially pronounced for the medium and coarse samples. The fine particles of both samples showed similar behaviour showing that the drag of gas was similarly efficient in deflecting particles laterally.

The results of our experiments suggest that the direction of volcanic ejecta is governed by vent geometry and the pressure at the vent exit. In case of a variable vent exit height, P_vent_ > P_atmosphere_ causes skewed ejection towards the lower vent side. In case of a funnel-shaped vent, the pressure at the vent quickly decreases to P_vent_ < P_atmosphere_ where the ejection is shifted towards the higher vent side. The same directional bias can be achieved through vents that have a varying vent divergence. However, pressure setting at the vent exit seems to have a lesser effect in this case. This relationship might allow a rough estimation of the pressure setting at the vent based on the observation of inclined jets if we have knowledge about the vent geometry and can exclude other factors influencing the inclination.

### Particle ejection velocity

The complex vents used in this study generated substantial variability in the velocity of particles tracked at two vent sides. The same variability was not observed in studies with symmetrical vent geometries (Cigala et al. 2017). Moreover, particle density was a major controlling parameter on ejection velocity (higher velocity for LSB than SL particles), while the particle size (of the particle size fractions that allowed particle tracking) only had a minor impact on velocity. Still, the highest velocity values were measured for medium particles.

The difference in particle velocity on either side of the vent was a consequence of the complex vent geometries. In experiments with the *cyl30* and *fun30* geometry, we measured a higher particle velocity on the left (lower) than on the opposing side (Table 3). This was only visible for the LSB particles since they were coupled sufficiently to still be affected by the unconfined gas flow. As the *fun30* vent decompressed more efficiently, the flow was only able to further affect the medium sized LSB particles in experiments with 15 MPa. Experiments with the *S1* geometry exhibited a uniform velocity distribution for SL particles for 8 and 15 MPa, whereas LSB particles were ejected faster on the right (more diverging) side of the vent. As a consequence of the asymmetric divergence angle of *S1*, the right (more diverging) side of the vent had a higher *M* and accordingly, higher gas velocities were reached on this side. Within the conduit, the acceleration can be assumed to be uniform and unilateral, but once the gas and particles reached the diverging section, the gas was able to accelerate stronger on ~ 40° side. Coupling between the gas and LSB particles was sufficient enough to be reflected by the high velocities of the particles, whereas the inertia of the SL particles prevented them from being fully accelerated by the gas in the diverging sections of the vents.

These observations can be interpreted when considering calculated non-dimensional fluid dynamic parameters based on the starting conditions of the experiments. We stress again that those values can only be regarded as conservative upper values as the impulsive nature of the experiments and the comparatively small autoclave volume caused highly dynamic conditions with only short periods during which a jet can be considered quasi-static (Peña Fernández et al. 2020). Only for the fine particles, St was close to 1, meaning that initially vertically and later additionally horizontally expanding gas allowed for more efficient acceleration within the autoclave and deflection (during the starting phase of particle ejection) above the vent. Since St was > 1 for medium and coarse particles of both densities, lateral deflection above the vent could be observed to a lesser degree. The particles followed trajectories dominated by inertia. While the gas flow likely started deceleration at or shortly after leaving the vent, the particle’s inertia prevented measurable deceleration in our field of view. The *fun30* vent geometry exhibited a higher ejection velocity than the other vent geometries. The higher exit-to-critical-area ratio and the higher M facilitated faster gas velocities than in experiments with the *cyl30* vent. According to fluid dynamic theory (Saad 1985), the *S1* vent with an even higher M should have produced a higher velocity. However, this was highly dependent on the exit pressure. A certain minimum pressure is required to positively correlate the exit-to-critical-area ratio and the M at the exit (Cigala et al. 2017). It seemed that the pressure had already dropped below the required minimum pressure as the particles arrived at the vent exit preventing a higher particle velocity.

### Linking experiments to volcanic hazards

The experiments with the *S1* geometry provide a proof of concept for incorporating novel techniques like UAV photogrammetry and 3D printing into the conception of experiments by bringing “real” volcanic geometries into the laboratory. A combination of high resolution, high-speed observations of the near-vent dynamics of volcanic explosions and scaled laboratory experiments utilising the associated “real” geometry can ultimately lead to establishing the link between observable features and the shallow subsurface initial conditions.

Although the setup of the experiments presented in this study did not allow observations beyond the near-vent region, the impact of complex vent geometries on gas-particle ejection can be compared with explosive volcanic eruptions by looking at field observations and published studies (e.g. Andrews and Gardner 2009; Jessop et al. 2016; Jessop and Jellinek 2014; Lagmay et al. 1999; Lherm and Jellinek 2019; Solovitz et al. 2014). Since our experiments produce starting jets, the dynamical evolution of pressure conditions more appropriately resembles the dynamics of individual volcanic eruption pulses or transient eruptions in contrast to sustained jets that are often assumed in numerical models and experiments. The use of real particles enables fluid-particle and particle–particle interactions which cannot be observed when using a pseudogas approach or a model solely accounting for two-way coupling.

Vent geometry is one of the prime factors controlling the initial ejection of pyroclasts. The particles used in the present study showed a variable degree of coupling as a function of size and density and different ratios between particle size and conduit diameter (Table 1), mimicking a wide range of volcanic ejecta. The gas flow initially accelerated the largest particles but they soon decoupled and continued on inertia-controlled ballistic trajectories. The (asymmetric) vent geometry thereby controlled the maximum ejection angle and velocity. In nature, the initial trajectory of volcanic ballistic projectiles directly results from vent and/or crater geometry, explosion depth, conduit inclination and secondary effects (e.g. vent coverage or clogging, presence of a high viscosity layer). The following trajectory is then controlled by a plethora of complex factors like drag forces, altitude, the surrounding expanding gas, in-flight particle collisions and particle deformation (e.g. Bower and Woods 1996; Fagents and Wilson 1993; Gaudin et al. 2016; Saunderson 2008; Sherwood 1967; Taddeucci et al. 2017; Vanderkluysen et al. 2012; Wilson 1972). However, the prime causes affecting the maximum travelling distance are ejection angle and velocity.

The asymmetric particle ejection angles and velocities can alter air entrainment in the jet asymmetrically. Trajectories that deviate from a vertical ejection can change the shape and size of entrainment eddies, increasing the penetration distance of the eddy compared to vertical trajectories (Jessop and Jellinek 2014). This effect might be especially strong for weakly coupled particles since their trajectories disturb the rotational motion of the eddies, increasing mixing rates and entrainment at the boundary layer (Lherm and Jellinek 2019). The experiments described by Jessop and Jellinek (2014) and Lherm and Jellinek (2019) describe the particle ejection into a water-filled tank describing a different regime that might not allow a direct comparison to the compressible regime of our experiments. Solovitz et al. [2014] observed an asymmetric ejection of the solid and fluid phases in experiments with erodible vents and gas-particle jets. They suggested that this asymmetric ejection may lead to partial fountain collapse. When a jet fails to entrain sufficient air to decrease its density below ambient levels, the asymmetry of the jet in the near vent region can lead to a preferential directionality of PDCs. In nature, collapse directions were linked to vent/crater asymmetry on several occasions, e.g. Mount St. Helens 1980 (Andrews and Gardner 2009), Mayon 1988 (Lagmay et al. 1999), Soufrière Hills 2010 (Cole et al. 2015) and Chaiten 2008–2009 (Major et al. 2013).

**Fig. 7:**
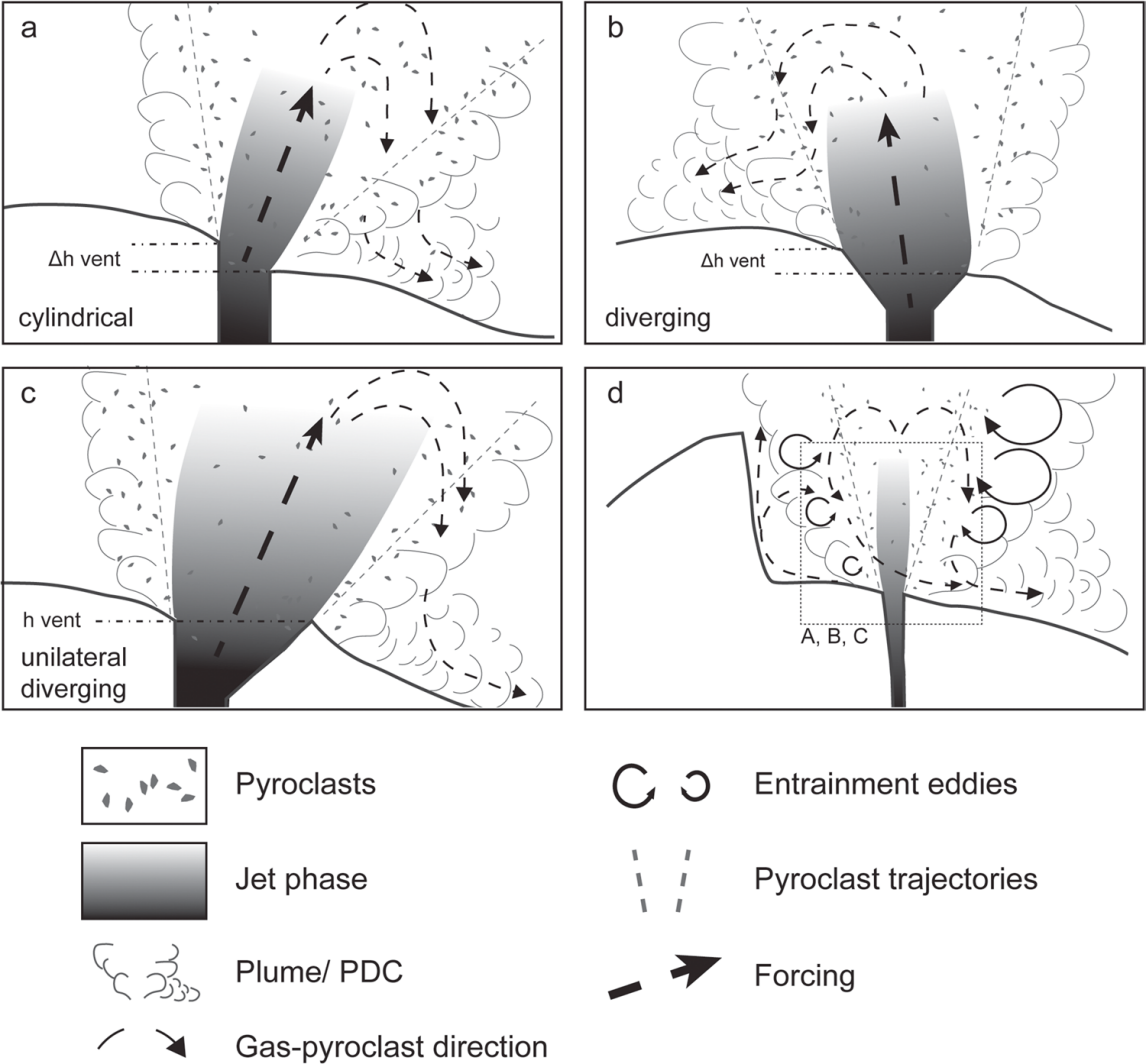
Sketch of possible column collapse scenarios. In **a** and **b**, the characteristic asymmetry element is the different vent exit height. The difference of the inner vent geometry (cylindrical and diverging) governs the inclination of the jet as a result of the decompression efficiency. In **c**, the asymmetric divergence describes the vent geometry. **d** Shows a larger field of view including the surrounding topography

**Fig. 9:**
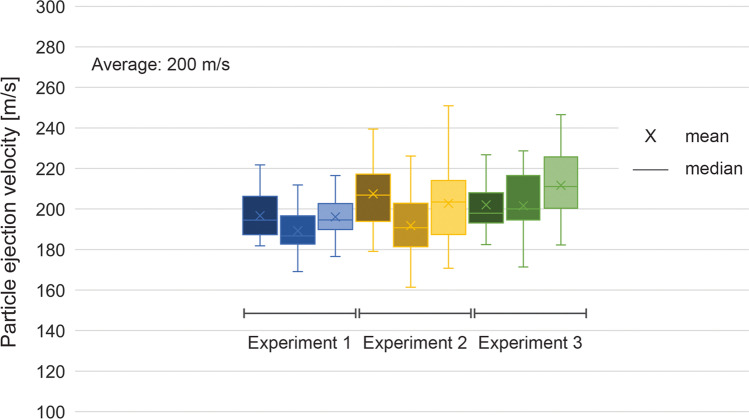
Test of reproducibility of particle velocity measurement. Three repetitions of the same experimental conditions (*fun30*, SL 1–2 mm, 15 MPa) were analysed three times each at the same time after t_0_. Every analysis was conducted independently and 25 particles were measured during each analysis. The 25 particles were selected unbiased; hence, it is possible that some particles were measured in multiple analysis. The box shows the quartiles calculated with an exclusive median. The cross marks the mean value and the line in the box marks the median. The whiskers show the minimum and maximum measured velocities

There are different mechanisms of how the vent and/or crater asymmetry can influence the direction of (partial) collapse of eruption columns. In Fig. 7a and b, for example, the emitted gas-pyroclast jets are tilted as result of the vent geometry. The (partial) collapse of inclined eruption columns — either due to vent asymmetry or an inclined conduit — will cause locally concentrated fallout and a preferential direction of PDCs. The link between inclined jets and vent asymmetry was demonstrated numerically (Lagmay et al. 1999) and experimentally (Schmid et al. 2020) and in the present study. This link is especially relevant for supersonic jets (Sim and Ogden 2012), where jet conditions (underexpanded/overexpanded) determine whether the jet is inclined towards the high or low side of the vent. For supersonic underexpanded jets, the preferred collapse direction is to the lowest side of the vent (Fig. 7a), while a supersonic overexpanded jet will focus the collapse towards the highest side of the vent (Fig. 7b) (Lagmay et al. 1999; Schmid et al. 2020).

If the characteristic asymmetry element is a varying divergence angle instead of a high and low vent exit side, the preferred direction for volcanic fallout and PDCs will be towards the more diverging side (Fig. 7c)) as a result of the asymmetric particle distribution. In the experiments presented in this study, the observation of asymmetric particle spreading angle suggests this behaviour.

In addition to vent geometry, the collapse direction can be affected by the asymmetry of the surrounding crater or topography. Jet and plume flow direction is partially restricted and consequentially deflected back and upwards, locally increasing the bulk density (Fig. 7d). The physical barrier might also limit air entrainment and restrict column radius, which leads to asymmetric column growth. A partial column collapses and directed PDCs due to asymmetric crater geometry was described by Andrews and Gardner (2009) for the 1980s eruption of Mount St. Helens. The dynamics of the experiments presented in this study do not permit an analysis of the criteria for column collapse. Hence, we cannot state whether the utilised complex vent geometries promote collapse over a symmetric geometry. However, based on:

1. Our experimental observations of particle trajectories and jet inclination,
2. Direct eruption observations, as well as
3. Published numerical models and experimental results, we suggest:

The asymmetric gas-particle jet spreading angles can initially encourage entrainment because of the increased surface area of the jet’s boundary layer and “increased penetration distance” (Jessop and Jellinek 2014) of the entrainment eddies until certain threshold conditions are reached (e.g. vent radius, mass eruption rate, ejection velocity). A comprehensive description of factors governing buoyant rise versus column collapse is beyond the scope of this study but have been described in numerous studies (Chojnicki et al. 2015; Dellino et al. 2014; Jessop et al. 2016; Koyaguchi et al. 2010; Lherm and Jellinek 2019; Neri et al. 2003; Saffaraval et al. 2012; Sparks et al. 1978; Suzuki et al. 2020; Woods 1988, 2010). We propose that an asymmetric vent and/or crater geometry can facilitate (i) heterogeneous air entrainment, (ii) an asymmetric distribution of volcanic ejecta and (iii) a preferential direction of ensuing PDCs in case of (partial) column collapse.

## Conclusion

The rapid decompression experiments performed in the present study investigated the link between vent geometry, particle size and density, and pressure and their impact on the eruption dynamics. In the laboratory, vent geometry determined the direction of the emitted gas-particle jet. The *cyl30* vent promoted the largest particle spreading angles, while the *fun30* vent exhibited the highest velocities. The *S1* geometry had the strongest asymmetry regarding the jet spreading angle. Both *cyl30* and *fun30* vents exhibited a larger spreading angle and a higher particle velocity (for LSB particles) on the left (lower) vent side than the right (higher) vent side. *S1* showed a larger spreading angle and faster particles (for LSB particle) on the side with the stronger divergence. In order of importance, the maximum particle spreading angle had:

- A negative correlation with particle size
- A negative correlation with particle density
- Positive correlation with experimental pressure
  The particle ejection velocity had:
- A negative correlation with particle density
- A positive correlation with experimental pressure
- A negative correlation with particle size

The results of the scaled laboratory experiments performed here show the significance of vent geometry and the major effect of asymmetry on the ejection of multiphase flows. These findings can be applied to interpret observable volcanic eruptions dynamics. The asymmetry of the vent and/or crater can impact areas affected by proximal and distal volcanic hazards. Furthermore, a comparison of the experimental data with field observations (Schmid et al. 2021) demonstrated the feasibility of using novel techniques to produce realistic vent geometries for laboratory experiments. The combination of UAV photogrammetry and additive 3D printing is a rapid and inexpensive way to utilise realistic volcanic vent geometries in scaled laboratory experiments.

Ultimately, we need increasingly complex experiments to explore the link between observable eruption dynamics and the underlying, concealed initial conditions that, to date, have remained beyond direct observation and measurements.

## Figures and Tables

**Fig. 8:**
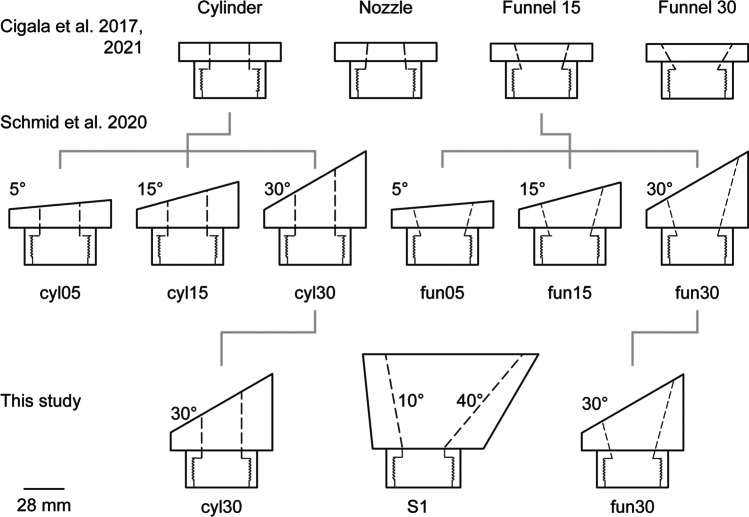
Evolution of experimental vent geometries used in rapid decompression experiments at Ludwig-Maximilians Universität, Munich. The vents in the first row were used by Cigala et al. (2017), (2021). They have a radial symmetry with varying exit diameters. Out of these four vents, two were selected and modified with a slanted exit plane (5, 15, 30°) to reduce the level of symmetry. These vents were used by Schmid et al. (2020) in gas-only experiments. The two vents with the strongest effect on the dynamics of gas jets were selected for the current study. They were complemented by a third vent based on the geometry of Stromboli’s S1 vent with an asymmetric divergence. All vents are sketched in a cross-sectional view. The upper part of *S1* is not to scale

**Fig. 10:**
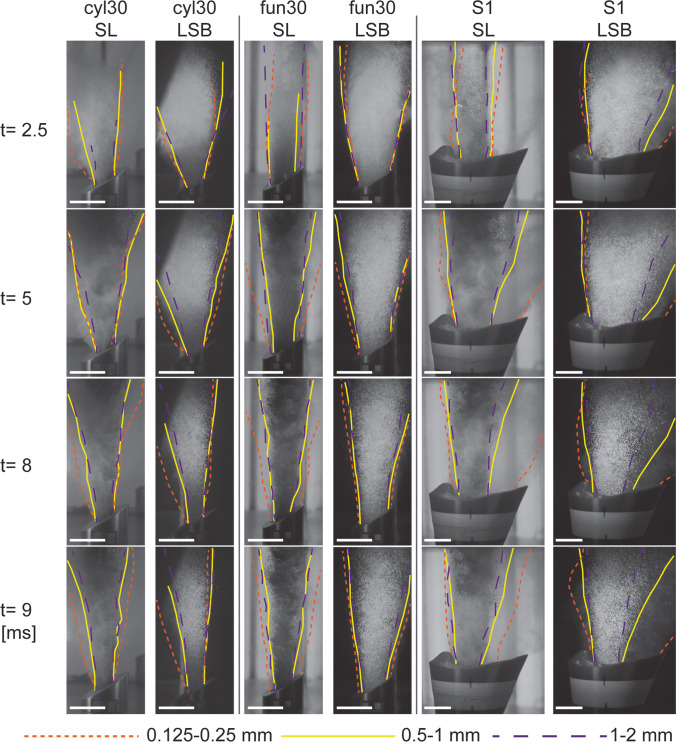
Time series of jet spreading angles at 8 MPa experimental pressure. Each vertical column shows the temporal evolution of particle ejection, with four rows at 2.5, 5, 8 and 9 ms after the onset of the gas ejection. The columns represent six different experimental conditions, using particles of two different densities (SL, LSB) and 3 different vent geometries (*cyl30*, *fun30*,* S1*). Colour lines mark the outlines of particle ejection. The dotted white line marks the centreline of the vent. For every individual stack of images, the corresponding image from the experiment with 8 MPa and 0.5–1 mm particles was taken as basis. t_0_ is defined as the onset of gas ejection and the scale bar shows 5 cm. This figure was created as shown in Fig. 3. Refer to Fig. 5 for the 15 MPa experiments

## Data Availability

Not applicable.

## Appendix

**Table 4:** Vent exit pressure (P_vent_) is affected by the starting pressure (P_start_) of the experiment and vent geometry. The pressure at the vent was measured with a *KISTLER* 601A pressure sensor just below the lower vent exit side. The *S1* vent was not equipped with a pressure sensor at the vent. P_start_ was measured at the top of the autoclave. Experiments where no pressure could be determined, either because no vent sensor was installed or a bad signal to noise ratio are marked as n.a

Experiment	0.125–0.25 mm		0.5–1 mm		1–2 mm	
	P_vent_ [MPa]	P_start_ [MPa]	P_vent_ [MPa]	P_start_ [MPa]	P_vent_ [MPa]	P_start_ [MPa]
cyl30						
SL, 8 MPa	n.a	8.4	n.a	n.a	2.3	8.2
SL, 15 MPa	n.a	15.5	2.7	15.2	0.7	15.3
LSB, 8 MPa	n.a	n.a	2.5	8.3	2.1	8.3
LSB, 15 MPa	n.a	n.a	4.0	15.2	3.8	15.4
fun30						
SL, 8 MPa	0.3	8.2	0.4	8.2	0.3	8.3
SL, 15 MPa	0.4	15.2	0.5	15.3	n.a	15.4
LSB, 8 MPa	0.8	8.1	0.3	8.0	0.4	8.0
LSB, 15 MPa	0.7	15.1	0.6	15.1	0.4	15.3
S1						
SL, 8 MPa	n.a	8.2	n.a	8.1	n.a	8.2
SL, 15 MPa	n.a	15.2	n.a	15.2	n.a	15.3
LSB, 8 MPa	n.a	8.2	n.a	8.3	n.a	8.2
LSB, 15 MPa	n.a	15.2	n.a	15.2	n.a	15.1

## References

[CR1] Alatorre-Ibargüengoitia MA, Scheu B, Dingwell DB (2011). Influence of the fragmentation process on the dynamics of Vulcanian eruptions: an experimental approach. Earth Planet Sci Lett.

[CR2] Alatorre-Ibargüengoitia MA, Morales-Iglesias H, Ramos-Hernández SG, Jon-Selvas J, Jiménez-Aguilar JM (2016). Hazard zoning for volcanic ballistic impacts at El Chichón Volcano (Mexico). Nat Hazards.

[CR3] Alidibirov M, Dingwell DB (1996). An experimental facility for the investigation of magma fragmentation by rapid decompression. Bull Volcanol.

[CR4] Alsoufi MS, Elsayed AE (2017). How surface roughness performance of printed parts manufactured by desktop FDM 3D printer with PLA+ is influenced by measuring direction. Am J Mech Eng.

[CR5] Andrews BJ, Gardner JE (2009). Turbulent dynamics of the 18 May 1980 Mount St Helens Eruption Column. Geol.

[CR6] Arciniega-Ceballos A, Alatorre-Ibargüengoitia M, Scheu B, Dingwell D (2015). Analysis of source characteristics of experimental gas burst and fragmentation explosions generated by rapid decompression of volcanic rocks. J Geophys Res Solid Earth.

[CR7] Bercovici D, Michaut C (2010). Two-phase dynamics of volcanic eruptions: compaction, compression and the conditions for choking. Geophys J Int.

[CR8] Blong RJ (2013). Volcanic hazards: a sourcebook on the effects of eruptions.

[CR9] Bower SM, Woods AW (1996). On the dispersal of clasts from volcanic craters during small explosive eruptions. J Volcanol Geotherm Res.

[CR10] Breard ECP (2014). Using the spatial distribution and lithology of ballistic blocks to interpret eruption sequence and dynamics: August 6 2012 Upper Te Maari eruption, New Zealand. J Volcanol Geotherm Res.

[CR11] Burgisser A, Bergantz GW, Breidenthal RE (2005). Addressing complexity in laboratory experiments: the scaling of dilute multiphase flows in magmatic systems. J Volcanol Geotherm Res.

[CR12] Capponi A, Taddeucci J, Scarlato P, Palladino DM (2016). Recycled ejecta modulating Strombolian explosions. Bull Volcanol.

[CR13] Carcano S, Bonaventura L, Ongaro TE, Neri A (2013). A semi-implicit, second-order-accurate numerical model for multiphase underexpanded volcanic jets. Geosci Model Dev.

[CR14] Carcano S, Esposti Ongaro T, Bonaventura L, Neri A (2014). Influence of grain-size distribution on the dynamics of underexpanded volcanic jets. J Volcanol Geotherm Res.

[CR15] Cerminara M, Esposti Ongaro T, Neri A (2016). Large Eddy Simulation of gas–particle kinematic decoupling and turbulent entrainment in volcanic plumes. J Volcanol Geotherm Res.

[CR16] Charbonnier SJ (2013). Evaluation of the impact of the 2010 pyroclastic density currents at Merapi volcano from high-resolution satellite imagery, field investigations and numerical simulations. J Volcanol Geotherm Res.

[CR17] Chojnicki K, Clarke A, Phillips J, Adrian R (2015). Rise dynamics of unsteady laboratory jets with implications for volcanic plumes. Earth Planet Sci Lett.

[CR18] Cigala V, Kueppers U, Peña Fernández JJ, Taddeucci J, Sesterhenn J, Dingwell DB (2017). The dynamics of volcanic jets: temporal evolution of particles exit velocity from shock-tube experiments. J Geophys Res Solid Earth.

[CR19] Cigala V, Kueppers U, Fernández JJP, Dingwell DB (2021). Linking gas and particle ejection dynamics to boundary conditions in scaled shock-tube experiments. Bull Volcanol.

[CR20] Clarke AB (2013). Unsteady explosive activity: Vulcanian eruptions.

[CR21] Cole P, Stinton A, Odbert H, Bonadonna C, Stewart R (2015). An inclined Vulcanian explosion and associated products. J Geol Soc.

[CR22] Dellino P (2014). Volcanic jets, plumes, and collapsing fountains: evidence from large-scale experiments, with particular emphasis on the entrainment rate. Bull Volcanol.

[CR23] Dempsey DE, Cronin SJ, Mei S, Kempa-Liehr AW (2020). Automatic precursor recognition and real-time forecasting of sudden explosive volcanic eruptions at Whakaari, New Zealand. Nat Commun.

[CR24] Dioguardi F, Dellino P, De Lorenzo S (2013). Integration of large-scale experiments and numerical simulations for the calibration of friction laws in volcanic conduit flows. J Volcanol Geotherm Res.

[CR25] Douillet GA, Rasmussen KR, Kueppers U, Castro DL, Merrison JP, Iversen JJ, Dingwell DB (2014). Saltation threshold for pyroclasts at various bedslopes: wind tunnel measurements. J Volcanol Geotherm Res.

[CR26] Druitt TH (1998). Pyroclastic density currents. Geol Soc London Spec Publ.

[CR27] Dürig T, Gudmundsson MT, Dellino P (2015). Reconstruction of the geometry of volcanic vents by trajectory tracking of fast ejecta - the case of the Eyjafjallajökull 2010 eruption (Iceland). Earth, Planets Space.

[CR28] Fagents SA, Wilson L (1993). Explosive volcanic eruptions—VII. The ranges of pyroclasts ejected in transient volcanic explosions. Geophys J Int.

[CR29] Fitzgerald RH (2014). The application of a calibrated 3D ballistic trajectory model to ballistic hazard assessments at Upper Te Maari, Tongariro. J Volcanol Geotherm Res.

[CR30] Gaudin D (2016). 3-D high-speed imaging of volcanic bomb trajectory in basaltic explosive eruptions. Geochem Geophys Geosyst.

[CR31] Gouhier M, Donnadieu F (2011). Systematic retrieval of ejecta velocities and gas fluxes at Etna volcano using L-Band Doppler radar. Bull Volcanol.

[CR32] Graettinger AH, Valentine GA, Sonder I (2015). Circum-crater variability of deposits from discrete, laterally and vertically migrating volcanic explosions: experimental evidence and field implications. J Volcanol Geotherm Res.

[CR33] Jessop DE, Jellinek AM (2014). Effects of particle mixtures and nozzle geometry on entrainment into volcanic jets. Geophys Res Lett.

[CR34] Jessop DE, Gilchrist J, Jellinek AM, Roche O (2016). Are eruptions from linear fissures and caldera ring dykes more likely to produce pyroclastic flows?. Earth Planet Sci Lett.

[CR35] Johnson JB, Watson LM, Palma JL, Dunham EM, Anderson JF (2018). Forecasting the eruption of an open-vent volcano using resonant infrasound tones. Geophys Res Lett.

[CR36] Kelfoun K, Harris A, Bontemps M, Labazuy P, Chausse F, Ripepe M, Donnadieu F (2020). A method for 3D reconstruction of volcanic bomb trajectories. Bull Volcanol.

[CR37] Kieffer SW (1989). Geologic nozzles. Rev Geophys.

[CR38] Kieffer SW, Sturtevant B (1984). Laboratory studies of volcanic jets. J Geophys Res Solid Earth.

[CR39] Koyaguchi T, Woods AW (1996). On the formation of eruption columns following explosive mixing of magma and surface-water. J Geophys Res Solid Earth.

[CR40] Koyaguchi T, Suzuki YJ, Kozono T (2010) Effects of the crater on eruption column dynamics. J Geophys Res Solid Earth 115:B07205. 10.1029/2009JB007146

[CR41] Kueppers U, Perugini D, Dingwell DB (2006). “Explosive energy” during volcanic eruptions from fractal analysis of pyroclasts. Earth Planet Sci Lett.

[CR42] Kueppers U, Scheu B, Spieler O, Dingwell DB (2006). Fragmentation efficiency of explosive volcanic eruptions: a study of experimentally generated pyroclasts. J Volcanol Geotherm Res.

[CR43] Lagmay MA, Pyle DM, Dade B, Oppenheimer C (1999). Control of crater morphology on flow path direction of Soufrière-type pyroclastic flows. J Geophys Res Solid Earth.

[CR44] Layana S (2020). Volcanic Anomalies monitoring System (VOLCANOMS), a low-cost volcanic monitoring system based on Landsat images. Remote Sensing.

[CR45] Lherm V, Jellinek AM (2019). Experimental constraints on the distinct effects of ash, lapilli, and larger pyroclasts on entrainment and mixing in volcanic plumes. Bull Volcanol.

[CR46] Lube G (2014). Dynamics of surges generated by hydrothermal blasts during the 6 August 2012 Te Maari eruption, Mt. Tongariro. New Zealand J Volcanol Geotherm Res.

[CR47] Lube G, Breard ECP, Esposti-Ongaro T, Dufek J, Brand B (2020). Multiphase flow behaviour and hazard prediction of pyroclastic density currents. Nat Rev Earth Environ.

[CR48] Major JJ, Pierson TC, Hobliltt RP, Moreno H (2013). Pyroclastic density currents associated with the 2008–2009 eruption of Chaitén Volcano (Chile): forest disturbances, deposits, and dynamics. Andean Geol.

[CR49] Meijering E, Dzyubachyk O, Smal I (2012). Methods for cell and particle tracking. Methods Enzymol.

[CR50] Montanaro C, Scheu B, Mayer K, Orsi G, Moretti R, Isaia R, Dingwell DB (2016). Experimental investigations on the explosivity of steam-driven eruptions: a case study of Solfatara volcano (Campi Flegrei). J Geophys Res Solid Earth.

[CR51] Neri A, Esposti OT, Macedonio G, Gidaspow D (2003) Multiparticle simulation of collapsing volcanic columns and pyroclastic flow. J Geophys Res Solid Earth 108:B4. 10.1029/2001JB000508

[CR52] Ogden DE (2011). Fluid dynamics in explosive volcanic vents and craters. Earth Planet Sci Lett.

[CR53] Oswatitsch K (1952). Gasdynamik.

[CR54] Peña Fernández JJ, Cigala V, Kueppers U, Sesterhenn J (2020). Acoustic analysis of starting jets in an anechoic chamber - implications for volcano monitoring. Sci Rep.

[CR55] Saad MA (1985) Compressible fluid flow. Prentice-Hall, Inc, Englewood Cliffs, p 570

[CR56] Saffaraval F, Solovitz SA, Ogden DE, Mastin LG (2012) Impact of reduced near‐field entrainment of overpressured volcanic jets on plume development. J Geophys Res Solid Earth 117:B05209. 10.1029/2011JB008862

[CR57] Salvatore V et al (2018) Parameterizing multi-vent activity at Stromboli Volcano (Aeolian Islands, Italy). Bull Volcanol 80:64. 10.1007/s00445-018-1239-8

[CR58] Saunderson HC (2008). Equations of motion and ballistic paths of volcanic ejecta. Comput Geosci.

[CR59] Scharff L, Hort M, Varley N (2015) Pulsed Vulcanian explosions: a characterization of eruption dynamics using Doppler radar. Geology 43(11). 10.1130/G36705.1

[CR60] Schindelin J (2012). Fiji: an open-source platform for biological-image analysis. Nat Methods.

[CR61] Schmid M, Kueppers U, Cigala V, Sesterhenn J, Dingwell DB (2020). Release characteristics of overpressurised gas from complex vents: implications for volcanic hazards. Bull Volcanol.

[CR62] Schmid M, Kueppers U, Civico R, Ricci T, Taddeucci J, Dingwell DB (2021). Characterising vent and crater shape changes at Stromboli: implications for risk areas. Volcanica.

[CR63] Sherwood AE (1967). Effect of air drag on particles ejected during explosive cratering. J Geophys Res.

[CR64] Sim S, Ogden D (2012) Effects of vent asymmetry on explosive eruptions. In: AGU Fall Meeting Abstracts, V41B-2791. 2012AGUFM.V41B2791S

[CR65] Solovitz SA, Ogden DE, Kim D, Kim SY (2014). Coupled fluid and solid evolution in analogue volcanic vents. J Geophys Res Solid Earth.

[CR66] Sparks R, Wilson L, Hulme G (1978). Theoretical modeling of the generation, movement, and emplacement of pyroclastic flows by column collapse. J Geophys Res Solid Earth.

[CR67] Spieler O, Kennedy B, Kueppers U, Dingwell DB, Scheu B, Taddeucci J (2004). The fragmentation threshold of pyroclastic rocks. Earth Planet Sci Lett.

[CR68] Sulpizio R, Dellino P, Doronzo DM, Sarocchi D (2014). Pyroclastic density currents: state of the art and perspectives. J Volcanol Geotherm Res.

[CR69] Suzuki YJ, Costa A, Koyaguchi T (2020) Control of vent geometry on the fluid dynamics of volcanic plumes: insights from numerical simulations. Geophys Res Lett 47:10. 10.1029/2020GL087038

[CR70] Taddeucci J, Scarlato P, Capponi A, Del Bello E, Cimarelli C, Palladino DM, Kueppers U (2012) High‐speed imaging of Strombolian explosions: the ejection velocity of pyroclasts. Geophys Res Lett 39:2. 10.1029/2011GL050404

[CR71] Taddeucci J, Valentine GA, Sonder I, White J, Ross P-S, Scarlato P (2013) The effect of pre-existing craters on the initial development of explosive volcanic eruptions: an experimental investigation. Geophys Res Lett 40:3. 10.1002/grl.50176

[CR72] Taddeucci J, Alatorre-Ibargüengoitia MA, Cruz-Vázquez O, Del Bello E, Scarlato P, Ricci T (2017). In-flight dynamics of volcanic ballistic projectiles. Rev Geophys.

[CR73] Valentine GA (1998) Eruption column physics. In: Freundt A, Rosi M (eds) From magma to tephra. Elsevier, Amsterdam, pp 91–138

[CR74] Vanderkluysen L, Harris AJL, Kelfoun K, Bonadonna C, Ripepe M (2012). Bombs behaving badly: unexpected trajectories and cooling of volcanic projectiles. Bull Volcanol.

[CR75] Williams GT, Kennedy BM, Wilson TM, Fitzgerald RH, Tsunematsu K, Teissier A (2017). Buildings vs. ballistics: quantifying the vulnerability of buildings to volcanic ballistic impacts using field studies and pneumatic cannon experiments. J Volcanol Geotherm Res.

[CR76] Wilson L (1972). Explosive volcanic eruptions-II the atmospheric trajectories of pyroclasts. Geophys J Int.

[CR77] Wilson L, Head JW (1981). Ascent and eruption of basaltic magma on the Earth and Moon. J Geophys Res Solid Earth.

[CR78] Wilson L, Sparks RSJ, Walker GP (1980). Explosive volcanic eruptions—IV. The control of magma properties and conduit geometry on eruption column behaviour. Geophys J Int.

[CR79] Woods AW (1988). The fluid dynamics and thermodynamics of eruption columns. Bull Volcanol.

[CR80] Woods AW (2010). Turbulent plumes in nature. Annu Rev Fluid Mech.

[CR81] Woods AW, Bower SM (1995). The decompression of volcanic jets in a crater during explosive volcanic eruptions. Earth Planet Sci Lett.

[CR82] Zanon V, Neri M, Pecora E (2009). Interpretation of data from the monitoring thermal camera of Stromboli volcano (Aeolian Islands, Italy). Geol Mag.

